# How to Use Macrophages Against Cancer

**DOI:** 10.3390/cells13231948

**Published:** 2024-11-23

**Authors:** Jacek Kuźnicki, Natalia Janicka, Barbara Białynicka-Birula, Wojciech Kuźnicki, Hanna Chorążyczewska, Iwona Deszcz, Julita Kulbacka

**Affiliations:** 1 Students Scientific Group No.148, Faculty of Medicine, Wroclaw Medical University, Borowska 211A, 50-556 Wroclaw, Poland; jacek.kuznicki@student.umw.edu.pl (J.K.); bewa.birula@gmail.com (B.B.-B.); hanna.chorazyczewska@student.umw.edu.pl (H.C.); 2 Students Scientific Group No.148, Faculty of Pharmacy, Wroclaw Medical University, Borowska 211A, 50-556 Wroclaw, Poland; natalia.janicka@student.umw.edu.pl; 3 Department of External Beam Radiotherapy, Nicolaus Copernicus Multidisciplinary Centre for Oncology and Traumatology, Pabianicka 62, 93-513 Łódź, Poland; w.kuznicki@kopernik.lodz.pl; 4 Department of Immunopathology and Molecular Biology, Faculty of Pharmacy, Wroclaw Medical University, Borowska 211A, 50-556 Wroclaw, Poland; iwona.deszcz@umw.edu.pl; 5 Department of Molecular and Cellular Biology, Faculty of Pharmacy, Wroclaw Medical University, Borowska 211A, 50-556 Wroclaw, Poland; 6 Department of Immunology and Bioelectrochemistry, State Research Institute Centre for Innovative Medicine Santariškių g. 5, LT-08406 Vilnius, Lithuania

**Keywords:** tumor-associated macrophages (TAMs), cancer immunotherapy, tumor microenvironment (TME), combined cancer therapies, TAM subtype impact

## Abstract

Numerous studies have demonstrated the significant influence of immune cells on cancer development and treatment. This study specifically examines tumor-associated macrophages (TAMs), detailing their characteristics and roles in tumorigenesis and analyzing the impact of the ratio of TAM subtypes on patient survival and prognosis. It is established that TAMs interact with immunotherapy, radiotherapy, and chemotherapy, thereby influencing the efficacy of these treatments. Emerging therapies are explored, such as the use of nanoparticles (NPs) for drug delivery to target TAMs and modify the tumor microenvironment (TME). Additionally, novel anticancer strategies like the use of chimeric antigen receptor macrophages (CAR-Ms) show promising results. Investigations into the training of macrophages using magnetic fields, plasma stimulation, and electroporation are also discussed. Finally, this study presents prospects for the combination of TAM-based therapies for enhanced cancer treatment outcomes.

## 1. Concept and Functions of Tumor-Associated Macrophages

The tumor microenvironment (TME) is a dynamic network where cancer cells induce molecular, cellular, and structural changes in the surrounding tissues, promoting their growth, development, and survival. The tumor extracellular matrix (ECM), composed of hyaluronan, elastin, fibronectin, laminin, glycoproteins, and proteoglycans, integrates cancer cells with a wide array of immune cells, stromal cells, and blood vessels, forming a complex three-dimensional structure [1,2]. Chronic inflammation accompanying cancer formation leads to the emergence of both adaptive and innate immune cells in the TME [3]. Macrophages, key cells of the adaptive immune response, perform a range of functions, including tissue repair and the regulation of homeostasis and immune activity. Within the TME, macrophages play a significant role as tumor-associated macrophages (TAMs) and can account for up to 30% of the tumor mass [4]. TAMs inhibit the immune response and participate in tumor initiation, progression, angiogenesis, and metastasis [5,6,7]. Data also indicate that TAMs play a role in chemotherapy resistance and reduce the effectiveness of immunotherapy [7,8,9].

### 1.1. TAMs in Tumorigenesis

TAMs, through a huge cross-section of cytokines, chemokines, growth factors, inflammatory substrates, and enzymes, affect the growth of cancer cells and inhibit T-cell activity. Numerous studies have confirmed that TAMs markedly enhance tumor progression [10,11]. In the early stages of cancer, M1 TAMs are present, participating in the inflammatory response and expressing many pro-inflammatory cytokines such as tumor necrosis factor (TNF)-α, interleukin-2 (IL-2), IL-6, IL-12, IL-23, nitric oxide (NO) and reactive oxygen species (ROS). In later stages, M2 TAMs predominate. Both phenotypes can transform each other in the TME, which may have clinical implications in oncology [12]. In most cancers, TAMs polarize mainly to the M2 phenotype, which has a pro-tumor effect [13,14]. Studies have confirmed the key role of M2 macrophages in initiating and maintaining the angiogenic process [15]. Hypoxia occurring in tumor areas leads to the expression of inflammatory molecules such as IL-4 or IL-10, resulting in an influx of macrophages and their polarization to the M2 phenotype [16]. Various pro-angiogenic cytokines and growth factors, like VEGF, TNFα, IL-8, and bFGF, are involved in the mechanisms of new blood vessel formation, as well as enzymes that regulate angiogenesis, which may include matrix metalloproteinases (MMPs) and cyclooxygenase-2 (COX-2) [17]. TAMs are involved in every step of metastasis. Impacts on invasion, vascularization, intravasation, extravasation, niche formation before metastasis, and the protection and survival of circulating tumor cells are noted [11]. Many studies have clarified the mechanisms underlying the migration and invasion of cancer cells involving TAMs, especially M2 [18,19,20,21,22].

### 1.2. M1/M2 Ratio and Impact on Prognosis

TAMs, due to their tumor-stimulating and metastatic effects, are a valuable prognostic factor. However, their association with the cancer stage depends on many coexisting factors [23]. For many types of cancer, including breast, bladder, prostate, and head and neck cancers, as well as glioma and melanoma, the degree of malignancy and poor prognosis correlate with a high density of TAMs [24]. In contrast, for colorectal cancer, a huge number of TAMs leads to longer patient survival [25]. The impact of TAMs on patient prognosis depends mainly on the type of macrophages in the tumor [26]. TAMs with surface expression of specific molecules may also be important in clinical observations. For example, in multiple myeloma, an unfavorable prognosis for a patient is determined by a large number of CD163+ TAMs [27]. In contrast, in gastric cancer, survival can be predicted by the presence of CD163+ macrophages in combination with CD66b+ neutrophils [28]. It is worth noting that it is not the total number of TAMs that indicates the tumor stage but the ratio of M1- to M2-polarized TAMs [29,30]. A high M1/M2 ratio mainly reflects a positive outcome and the prolongation the patient’s life. Similarly, a low M1/M2 ratio mostly determines a poor prognosis and a worse response to treatment. Therefore, this ratio is an accurate prognostic factor that has clinical applications in determining the length of patients’ survival [31,32,33].

## 2. Macrophage-Related Anticancer Tools

### 2.1. Chemo-, Immuno-, and Radiotherapy

Macrophages play a crucial role in the interactions between cancer therapy and the immune system. Different cancer therapies, such as immunotherapy, chemotherapy, and radiotherapy, have been shown to modulate macrophage function and induce antitumor responses. However, macrophages can also contribute to tumor resistance to chemo- and radiotherapy by promoting tumor cell survival and proliferation. Understanding the interactions between macrophages and various cancer therapies is crucial for developing more effective cancer treatments.

#### 2.1.1. Immunotherapy

During anticancer therapy, TAMs either support treatment or reduce its effectiveness. In order to minimize the negative and maximize the positive impact of TAMs on anticancer therapy, strategies have been developed to modulate those cells. Different approaches have been proposed to modulate TAMs, including the depletion of TAMs, inhibition of circulating monocyte recruitment into the tumor, blockade of the M2 phenotype, and enhanced activation of M1 macrophages or reprogramming of TAMs toward M1 macrophages [34].

Substances used for the depletion of TAMs include bisphosphonates, primarily clodronate and zoledronic acid. Bisphosphonates are often used to encapsulate liposomes for better drug delivery, and they have been proven to affect tumor cells directly [35]. In mice treated with clodrolip (liposome-mediated clodronate), there was a significant decrease in macrophage infiltration to the tumor site, and reduced bone metastasis was also observed [35]. Clodrolip inhibits tumor growth and depletes TAMs, and these effects can be enhanced by the systemic application of anti-VEGF antibodies, which exert antiangiogenic effects [36]. It can also be used to deplete macrophages present in the liver. Clodrolip allows for better efficiency in therapy combined with PTX-PLGA (paclitaxel-poly(lactic-co-glycolic acid) chemotherapy [37]. Combinations of either clodrolip or zoledronic acid with sorafenib are used in cases of metastatic liver cancer [35]. Zoledronic acid on its own has become an important component in the treatment of cancer [38]. It shows anticancer effects in prostate cancer treatment and reduces bone metastasis in animal models [39].

Another substance—trabectedin, also known as ET-743—has been approved by the FDA and has demonstrated a cytotoxic effect against TAMs while exhibiting antitumor activity [40]. It is an antitumor agent that selectively depletes mononuclear phagocytes in both blood and tumor tissues. It is primarily used to treat advanced soft tissue sarcoma that has metastasized to other parts of the body when other treatment options, like ifosfamide and anthracycline chemotherapies such as doxorubicin, have been ineffective or unsuitable. Trabectedin is also prescribed in combination with another chemotherapy drug called liposomal doxorubicin to treat ovarian cancer that has recurred [40].

One more substance known to deplete TAMs is osthole, a coumarin member isolated from *Cnidiummonnieri* (*Fructus Cnidii*). Osthole has been found to decrease M2 macrophages in pancreatic tumors. This effect is achieved through the inhibition of STAT6 and p-ERK1/2-C/EBP β [41]. CSF1-R inhibitors have been shown to effectively eliminate TAMs and halt tumor growth, angiogenesis, and metastasis. Specifically, BLZ945 is a highly selective small-molecule inhibitor of CSF1-R tyrosine kinase, leading to the depletion of TAMs while simultaneously increasing the number of cytotoxic (CD8+) lymphocytes in murine cervical and breast carcinoma models [34,42].

Another promising approach to TAM depletion is the activation of cytotoxic T lymphocytes, which can selectively target and eliminate macrophages. One such method involves modifying the gene sequence of legumain in order to enhance the effectiveness of immunization against it. This leads to reduced legumain maturation and impaired cellular localization [34]. Ultimately, this results in the elimination of TAMs dependent on CD4+ and CD8+ lymphocytes. The use of toxin-conjugated monoclonal antibodies (mAbs) and attenuated bacteria that target and kill macrophages represent other potential strategies for depleting TAMs [34].

The blocking of the M2 phenotype of macrophages can be accomplished by targeting transcription factors STAT3 and STAT6. Various inhibitors, including sorafenib, sunitinib, WP1066, and resveratrol, target STAT3, while 4-HPR, leflunomide, TMX264, and AS1217499 target STAT6. Resveratrol and fenretinide were found to inhibit M2 polarization of TAMs by decreasing STAT3 and STAT6 activity, respectively, resulting in tumor regression. Inhibitors of STAT6 activation, including TMC264 and AS1517499, have also been developed. Additionally, the small-molecule inhibitor of STAT3, WP1066, has shown potential in reversing immune tolerance in patients with malignant glioma [43].

In order to enhance the activation of M1 macrophages and repolarize TAMs into the M1 phenotype, a few methods have been developed. Such methods include stimulating STAT1 with IFNγ or vadimezan, activating AMPKα1 with metformin, using nuclear factor kappa B through toll-like receptor agonists such as imiquimod or CpG-ODNs, and PI3Kγ deletion. Inhibiting placental growth factor (PlGF) with HRG or C/EBPβ with PI3Kγ deletion can also lead to effective reprogramming of TAMs towards M1-like macrophages. Additionally, stimulating CD40 with monoclonal antibodies (mAbs) against CD40 converts TAMs from the M2 phenotype to M1 macrophages [34].

The combination of CD40 agonist with gemcitabine has shown promising results in promoting antitumor macrophages in pancreatic cancer patients. TLR agonists, anti-CD40 mAbs, and IL-10 mAbs can activate the NF-κB pathway to polarize macrophages towards an antitumor phenotype. Inhibition of NF-κB activity can also induce a tumoricidal phenotype in macrophages. Modulating STAT1 activity with cytokines like IFN-γ or GM-CSF can also promote an antitumor phenotype in macrophages. Tyrosine kinase inhibitors like sorafenib and sunitinib can inhibit STAT3 in macrophages and reverse the immunosuppressive cytokine profile of TAMs [43].

Drugs like histidine-rich glycoprotein, copper chelate, 5,6-dimethy XAA xanthenone-4-acetic acid, and vadimezan (ASA404) can suppress TAMs by targeting different functional properties of pro-tumor macrophages. These agents can be used as combinatorial therapies to effectively induce an antitumor phenotype in macrophages. Silibinin and proton pump inhibitors are other chemotherapeutic agents that can target different functional properties of pro-tumor macrophages. Overall, these strategies provide potential targets for the development of macrophage-related immunotherapies for patients with cancer [43].

Naturally occurring substances such as baicalein, a compound extracted from *Scutellaria baicalensis* root, have been shown to block TGF-β1 by inhibiting the PI3K/Akt pathway in M2 macrophages, thereby converting them into the M1 phenotype in breast cancer tissues [44]. Meanwhile, extracts from the root of *Panax notoginseng*, especially Ginsenoside Rb3, can promote the differentiation of M2 macrophages into the M1 type and provide protective functions against acute lung injury. Emodin, a natural anthraquinone derivative from Chinese herbs, can regulate both M1 and M2 phenotype programs by suppressing STAT6 and C/EBPβ signaling and restrain excessive M1 or M2 macrophages [41].

Other natural compounds, such as those isolated from *Briareum violaceum*, gorgonian *Pseudopterogorgia americana*, purine alkaloid homarine, and crustaceans of the order Decapoda, have also been found to have the potential to modulate macrophage polarization towards M1 or M2 phenotypes. Triterpene glycosides extracted from sea cucumbers have a pronounced immunomodulatory effect. They are capable of activating macrophages and polarizing them into the M1 phenotype by affecting the pathway mediated by purinergic P2 × 4 receptors, leading to increased cell adhesion, spreading, motility, lysosomal and bactericidal activity, pro-inflammatory cytokine release, and expression of iNOS, as well as increases in ROS and NO levels [41].

The recruitment of macrophages and myeloid cells into the tumor microenvironment can be prevented through the inhibition of CCL2/C–C chemokine receptor type 2 or CSF1/CSF1-R. Anti-CCL2 monoclonal antibodies such as carlumab can be used for that purpose through the inhibition of CCL2 synthesis using bindarit or trabectedin. Bindarit has been shown to reduce TAM and myeloid-derived suppressor cell infiltration in breast and prostate cancer models. Trabectedin is also used to inhibit monocyte recruitment and treat ovarian and breast cancer, as well as soft tissue sarcomas [34]. Both chemo- and radiotherapy are affected by TAMs. Therefore, studying the relationship between anticancer therapies and TAMs can lead to a better understanding of their effectiveness and optimization.

#### 2.1.2. Chemotherapy

**Figure 1:**
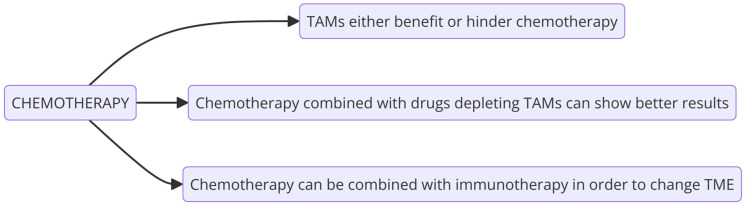
Interactions between chemotherapy and TAMs (prepared with Diagrams.helpful.dev, accessed on 9 September 2024).

Depending on different factors, TAMs either benefit or hinder chemotherapy (Figure 1). For example, doxorubicin (DOX) is a drug used in the treatment of solid tumors and hematological malignancies, including breast, bile duct, prostate, uterine, ovarian, esophageal, stomach, and liver tumors; childhood solid tumors; osteosarcomas; soft-tissue sarcomas; Kaposi’s sarcoma; acute myeloblastic and lymphoblastic leukemia; and Wilms tumors [45]. However, it has been reported that the therapeutic effects of DOX are affected by macrophages in both positive and negative ways. Macrophages can contribute to such effects through mechanisms of myeloid cell recruitment, differentiation into antigen-presenting cells (APCs), activation of immune responses, immunogenic tumor cell death, and myeloid-derived suppressor cell (MDSC) depletion, but they can also limit therapy response via misguided tissue repair [9].

Depending on the drugs used in therapy, TAMs exert different effects on cancer therapy. The therapeutic functions of drugs such as paclitaxel, etoposide, platinum, gemcitabine, and others are less effective because of the protective function of TAMs [9]. Because of that, chemotherapy has been combined with immunotherapy in order to change the tumor environment. Chemotherapy combined with drugs depleting TAMs can show better results [46] (Figure 1).

#### 2.1.3. Radiotherapy

After irradiation, macrophages are recruited to the affected site. Studies have shown that migrating macrophages strongly influence the development of radiation injury and the effectiveness of anticancer therapy. Radiation can change the phenotypes of macrophages migrating to the site depending on the time and total/fraction dose [47].

**Figure 2:**
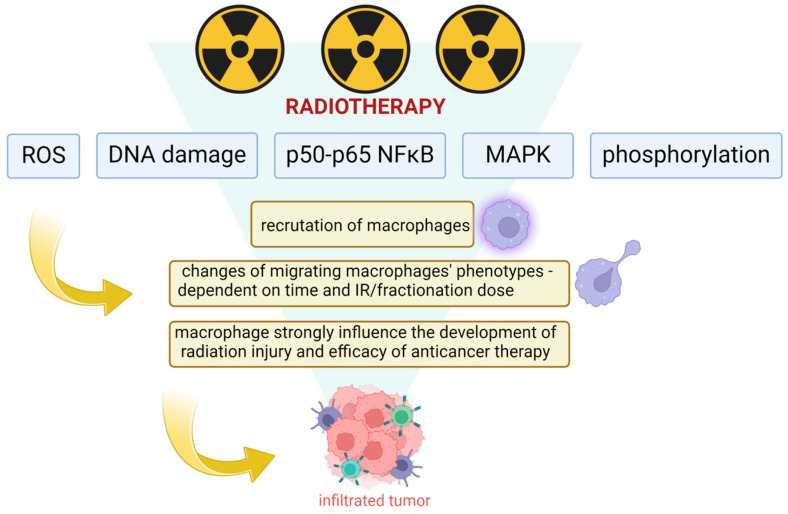
Radiotherapy and its effects on TAMs.

Radiotherapy affects TAMs through mechanisms such as ROS, DNA damage, p50–p65 NFκB activation, and MAPK phosphorylation (Figure 2). Genard et al. analyzed and summarized the effects of different fractionation doses on TAMs. Differences between the effects of doses of irradiation are due to the different mechanisms they trigger. For example, moderate-fraction doses (1–10 Gy) switch the NFκB subunit balance in the direction of the active homodimer, which correlates with the reprogramming of TAMs. High doses of irradiation induce apoptosis and switch the NFκB subunit balance in the direction of the inactive homodimer. Doses of localized radiation higher than 10 Gy and lower than 1 Gy promote M2 polarization. Only MDI (Moderate Doses Irradiation) (ranging from 1 to 10 Gy) of localized irradiation promotes eligible M1 polarization [34].

In another study, it was similarly reported that an irradiation dose of under 2 Gy increases the number of M2 TAMs in the tumor burden, while some TAMs also repolarize towards the M1 phenotype. Doses between of 4 10 Gy decrease M2-phenotypic traits but increase the overall number of macrophages. Higher doses of over 10 Gy increase the number of M2-phenotypic macrophages [48]. Therefore, MDI combined with chemo- or immunotherapies targeting macrophage reprogramming could also synergize their effects on tumor regression.

### 2.2. Nanoparticle-Targeted Drug Delivery

**Figure 3:**
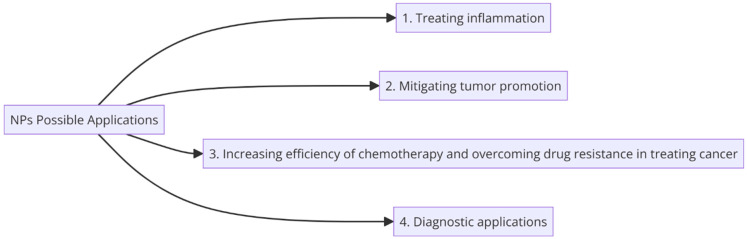
Possible applications of NPs (prepared with Diagrams.helpful.dev, accessed on 9 September 2024).

Nanoparticles (NPs) used in targeted drug delivery therapy act as agents that can be used to cure inflammation and cancer (Figure 3). The use of NPs is said to be an effective method for mitigating tumor promotion, in addition to increasing the efficiency of chemotherapy and overcome drug resistance the treatment of cancer. Traditional drugs are burdened by limitations such as non-specific distribution, potential toxicity, a lack of targeting capability, poor solubility in water, and low therapeutic indices. NPs transcend these limitations. However, it is crucial to design NPs in a way that enables them to be effective in real environments. Dynamics, pH, leaky vasculature, and hypoxia are properties of the TME that NPs used in cancer therapy have to be designed for [49].

One mechanism by which macrophages could be utilized in cancer drug delivery is through their ability to engulf and phagocytose foreign substances such as nanoparticles [50]. Nanoparticles can be designed to carry therapeutic agents within them; once taken up by macrophages, drugs can be released in the tumor microenvironment. Macrophages act as drug carriers and deliver therapeutic agents directly to cancer cells. In addition to the phagocytosis mechanism, it has been suggested that TAMs could be manipulated through phenotypic changes [51].

The two main objectives of NPs targeting TAMs are to deplete TAMs and reprogram them into the M1 phenotype. Using liposome-coated anti-CCR2, the inhibition of monocyte recruitment and decreases in the recruitment of primary and metastatic tumor lesions can be achieved. Chitosan-containing anti-CSF-1 interferes with the survival of TAMs by inducing apoptosis. Gold NPs also induce apoptosis. The use of TLR agonists and PD-1 blockers is another strategy used to reprogram TAMs. Such methods use silica NPs and carbon NPs. Polymers can be used to deliver IL-12 in order to induce M1 polarization [49].

An example of NPs modifying the TME and TAMs is found in iron oxide nanoparticles (IONPs). IONPs not only provide an efficient vehicle for antigen delivery but also reprogram TAMs toward an immunogenic phenotype through direct engagement and activation of immune response-related receptors like TLRs. They can be used as OVA (ovalbumin)-coated IONPs, which, among other properties, effectively prevent lung metastasis by OVA-expressing cells and activate macrophages [52]. Another study utilized arginine nanoparticles (Arg-NPs) as a vehicle to deliver CRISPR-Cas9 gene-editing machinery into cells to produce SIRP-α knockout macrophages, obstructing their binding to CD47 [41]. This led to a four-fold increase in the phagocytosis of human osteosarcoma U2OS cancer cells [41]. There is also interest in using macrophages as vehicles for the delivery of CAR-T cells [53].

NPs have diagnostic applications as well. Based on the type of NP, PET, CT, and MRI imaging modalities and their combinations can be used to detect the accumulation of NPs in macrophages [49]. Rodell et al. [54] proposed strategies for drug delivery in the screening and therapeutic re-education of TAMs. The strategy includes the development of a morphometric polarization screen for macrophage-polarizing agents that then allows for the identification of repolarizing drugs. Another step is the design of nanoparticles with drug-binding affinity and TAM avidity. The last step is the analysis of nanoparticle biodistribution and accumulation, as well as therapeutic efficacy, in TAMs. The study also concluded that TLR7/8 agonists (R848-resiquimod) are most efficient at re-educating TAMs in vitro [54].

### 2.3. Macrophages Engineered by CAR

CAR-Ms are human primary macrophages armed with transduced chimeric antigen receptors (CARs). Their abilities, such as phagocytosis of selective antigen-expressing tumor cells, cellular toxicity, secretion of pro-inflammatory factors, and the presentation of antigens to T cells, are important therapeutic properties in anticancer therapies [55]. With the help of myeloid cells, CAR-Ms can directly enter solid tumor sites and selectively kill antigen-expressing tumor cells [41,56]. Research on solid-tumor xenograft mouse models confirmed this by decreasing tumor burden and prolonging overall survival [56].

CAR-Ms can be produced on a large scale and are expandable, thanks to the fact that they can be generated from peripheral blood, iPSCs, or human leukemia monocytic cell line THP-1 [56,57]. An example of a proposed manufacturing process involves blood collection, monocyte isolation, ex vivo differentiation to macrophages, transduction with Adf535, cryopreservation in infusible media, and meticulous quality control [55].

Zhang J. et al. [58] successfully created hPSC (human pluripotent stem cell)- derived anti-GD2 CAR-Ms, which were effective in killing neuroblastoma in vivo and melanoma cells in vitro. Scientists generated CAR-Ms with the CRISPR-Cas9 gene editing method to integrate anti-GD2 CARs into the AAVS1 locus of hPSCs. Following that, a serum- and feeder-free differentiation protocol was established in order to generate CAR-Ms through the arterial endothelial-to-hematopoietic transition. The study presented the effectiveness of hPSC-derived CAR-Ms against solid tumor cells [58].

There are many advantages of using CAR-Ms. First of all, they possess the ability to phagocyte selective antigen-expressing tumor cells, as well as to migrate and infiltrate into an immunosuppressive tumor environment. Those abilities give CAR-Ms an edge over CAT-T and CAR-NK therapies. Secondly, CAR-Ms can also induce a phenotypic shift in M2 macrophages to the M1 type and activate T cells [56]. Other advantages include a low GvHD risk, resistance to the effects of immunosuppressive cytokines, the ability to present tumor antigens to Th1 cells, the ability to recruit leukocytes, and the ability to induce an adaptive immune response and produce anti-inflammatory factors. In the TME, AR-Ms also become an important source of matrix metalloproteinase (MMP), which degrades almost the entire ECM (extracellular matrix) [55,57]. Problems concerning CAR-M therapy are as follows. There is limited efficacy in CAR transduction. It requires differentiation into the M1 antitumor phenotype. Hypothetically, the use of CAR-Ms could lead to a cytokine release storm (CRS), and off-target toxicity.

Strategies were proposed by Maalej et al. [57] to increase the effectiveness of CAR-M therapy. Their three main objectives were to improve the bioengineering of CAR-Ms, enhance the antitumor activity of CAR-Ms, and enhance the trafficking and persistence within the immunosuppressive TME. Improving the bioengineering of CAR-Ms can be achieved by the use of modified lentiviral virions containing Vpx, the use of AD5f35 (adenovirus 5-fiber 35 vectors), and the use of MPEI (mannose-conjugated polyethyleneimine. To enhance antitumor activity, CAR-Ms can be incubated in vitro with MSLN-expressing ovarian (OVCAR3) and pancreatic (ASPC1) cancer cells in order to switch into the M1 phenotype. Finally, trafficking and persistence within the TME can be enhanced by the CAR-CD147 construct; the use of CCL19-expressing CAR-Ms; and combination therapies with anti-CD47, anti-CD20, and anti-TAA antibodies [57]. CAR-iMACs (CAR in iPSC-derived macrophages) could be the future of CAR-M therapy, as iPSCs (induced pluripotent stem cells) provide a vast number of easily engineered cells. These cells can be modified by toll-like receptor 4 intracellular toll/IL-1R, which improves the stability of the M1 phenotype [59]. The application of OMV (outer-membrane vesicles) has been proposed in order to enhance CAR-M therapy [60].

Clinical trials were performed on two mouse models of solid-tumor xenografts. The results indicated that a single infusion of CAR-Ms reduces the growth of human tumors and increases the survival of tumor-bearing animals [41]. Schepisi G et al. [61] evaluated three clinical trials concerning the CAR-M-based strategy in solid tumors conducted prior to December 2022. A Phase I clinical trial conducted by CARISMA Therapeutics Inc. (Philadelphia, PA, USA) (NCT04660929) involved the therapeutic use of anti-HER2 CAR-Ms in 18 cases of patients with relapsed or refractory HER2-overexpressing tumors. The second trial, MCY-M11 (NCT03608618; MaxCyte Inc., Gaithersburg, MD, USA), was performed on patients with relapsed/refractory ovarian cancer and peritoneal mesothelioma. The third trial (CARMA-2101; NCT05007379), an observational study, was conducted on organiods derived from 100 breast cancer patients. The activity of CAR-Ms was examined against those organoids, which were derived from either HER-negative, HER2-low, or HER2-positive breast cancer. The activity of CAR-Ms and non-modified macrophages was tested [61]. At the beginning of 2024, another early phase 1 trial (NCT06224738) was started, also investigating human anti-HER2 CAR-Ms but in advanced gastric cancer with peritoneal metastases (Table 1).

There are already proposals for possible combination therapies for CAR-Ms. Maalej et al. [57] proposed three combinations that enhance the phagocytic properties of CAR-Ms: (1) combination with anti-CD47 and anti-HER2 (trastuzumab), (2) combination with anti-CD47 and anti-CD20 (rituximab), and (3) combination with anti-PD-1 (anti-programmed cell death protein 1). They also proposed a CAR-M/CAR-T combination model while suggesting that combining CAR-Ms with CAR-NK cells or CAR-T cells is effective in enhancing their antitumor efficacy [57].

One study was published about a second-generation CAR-iMAC that shows higher efficacy than the first generation and could be used in treatments targeting solid tumors [62]. Another study reported that in animal models, CAR-iMAC exhibits antitumor function and improves the survival of pancreatic mouse models while being a safe treatment [63]. CAR-M strategies are listed in Table 2.

## 3. Methods of Training Macrophages Against Cancer Cells

### 3.1. Alternative Activation by Cytokines

Macrophages need to be activated in order to perform their function. The process is artificially divided into several steps: differentiation, priming, activation, and resolution [65]. As a result, many phenotypes of macrophages are ready to act in different environments. The outcome of the first phase is mostly based on the MCSF:GMCSF ratio and the number of lipoproteins and retinoic acids, whereas the results of the second step can be determined by many different cytokines [66]. The classical means of activation is through stimulation of the macrophages by interferon-gamma (IFN-γ) and tumor necrosis factor (TNF). These stimuli result in the differentiation of classically activated macrophages that participate in the inflammatory response. The other form of activated macrophages is regulatory macrophages, which are quite a heterogeneous group of cells characterized by high levels of IL-10 production. Finally, there are alternatively activated macrophages (AAMs). This phenotype is induced by the exposure of cells to IL-4 and IL-13 [67]. These cytokines are produced by T-helper 2 cells (Th2), mast cells, basophils, eosinophils, and NKT (Natural Killer T) cells. IL-33 and IL-25 are known to escalate the development of AAMs indirectly, acting via Th2 cells. IL-4 causes the upregulation of the mannose receptor, in contrast to the classical phenotype. Furthermore, the metabolism of arginine differs from the classical phenotype, in which upregulation of the induced nitric oxide synthase (iNOS) results in increased production of NO, which is an important antimicrobial agent. Instead, the activity of arginase is increased, which results in the production of more ornithine, a substrate for collagen and polyamine synthesis, serving as materials for the reconstruction of tissues in wound healing. Therefore, AAMs show little or no microbiocidal activity and are dedicated to rebuilding tissue after inflammation, similar to M2 macrophages.

### 3.2. Magnetic Field

The effects of magnetic fields (MFs) on cells have been intensely researched in recent years, creating new uses of magnetic fields in biomedical sciences [68]. The type and intensity of the effects of electromagnetic fields on cell biology mainly depend on the polarization produced by the fields. Electric or magnetic susceptibility is the quantitative value that says to what extent a specific source of polarization can polarize an object. The magnetic susceptibility is about 10^5^–10^6^ smaller than the electric susceptibility, which means the magnetic field does not affect the integrity of cells as much, not damaging the internal structures. This creates new opportunities for the development of therapies [69].

It has been proven that low-intensity magnetic fields (low frequencies up to 50 Hz) activate a mild pro-inflammatory response, whereas moderate- and high-intensity magnetic fields promote an immune response in the anti-inflammatory direction [70]. Specifically, macrophages and their behavior upon exposure to magnetic fields have also been investigated, as these cells play a crucial role in the immunological response. It was shown that the exposure of the macrophages to extremely low-frequency MFs causes an increase in the levels of IL-12 in the environment, which is a signature marker of M1 macrophages, which suggests that such stimulation promotes the pro-inflammatory phenotype and, therefore, would be a great tool in cancer therapy. This method has proven to be effective in treating the mouse H22 hepatocellular carcinoma. Artificially induced macrophages are known to degrade the tumor cells [71]. Moreover, it was proven earlier that the production of radical oxygen species and the intensity of phagocytosis increase significantly under ELFMFs (extremely low-frequency magnetic fields) [72,73]. The effects of HGMF (high-gradient magnetic fields) on macrophages are the opposite. Macrophages, like other eukaryotic cells, have a cytoskeleton consisting of actin. These filaments enable phagocytic cells to migrate and, therefore, make them apt to perform their duties. The effects of HGMFs on the macrophages, specifically on their cytoskeleton, were investigated thoroughly in [69]. In one of the studies, M0 macrophages were grown on a pair of magnets. Such exposure resulted in the elongation of the macrophages due to the disruption of the structure of the actin filaments. These results are similar to the outcome of either pharmacological or genetical interference with the RhoA pathway; however, the mechanism of the process is different. Under normal conditions, the RhoA pathway leads to the activation of immune cells, resulting in migration to the endangered spot and facilitating the internalization of pathogens.

When disturbed, macrophages cannot differentiate from the M1 phenotype properly [74]. It was also proven that M0 macrophages grown on the magnets expressed Arg-1, a marker of M2 macrophages, and no group expressed iNOS [70]. It was also observed that the macrophages aligned with the magnetic force pattern in rows as if they followed each other. Such an arrangement is also noted for fibroblasts during wound healing, which suggests that the anti-inflammatory phenotype of macrophages also falls into the scheme of one cell guiding the rest, although more tests need to be performed. Finally, the aggregation of the TRPM2 channels was excessive in some parts of the cell membrane, whereas in some areas, there was not a sufficient number of channels, which caused impairment of the transport of calcium ions. Calcium ions are an important factor vital for actin polymerization, so the process could not occur correctly. These results suggest that in the future, the magnetic field might be used more commonly for RhoA pathway interference in order to change the phenotype of the macrophages.

**Figure 4:**
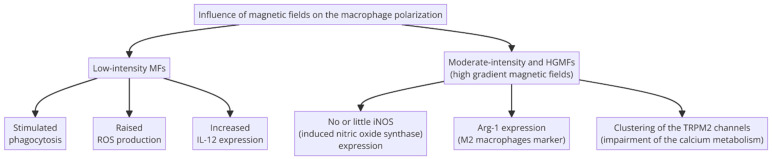
The influence of magnetic fields on macrophage polarization [69,77] (prepared with Diagrams.helpful.dev, accessed on 9 September 2024).

After some modification, macrophages can also become powerful, easy-to-guide antitumor weapons. Recently, the idea of immunobots has been presented in the scientific environment. Macrophages are incubated with FePt-marked and with bacterial lipopolysaccharide (LPS), which leads to the phagocytosis of microorganisms by the macrophages. Then, they are placed in the vicinity of the neoplastic cells. As a result of incubation with the bacteria, when an external magnetic field is appropriately applied, the macrophages move in the desired direction (in this case, towards cancer cells), having a detrimental effect on the tumor. It is due to stimulation by the bacterial LPS that the immunobots show M1 characteristics, secreting TNF-α, IL-6, IL-12, and M1 markers like iNOS and CD80. It has been proven that designed immunobots reduce the number of tumor cells in urinary bladder carcinoma. This method presents many assets, such as not activating the host immune system [75]. However, there are some limitations to this technique that are being addressed now. Some immunobots have difficulty localizing the macrophages and actuating them, and they struggle to find a proper imaging technique to observe the progress of therapy. As reported recently in [76], the use of Janus particles and multimodal imaging might be a solution to these problems. The authors proposed an approach of integration of many non-invasive imaging techniques (optoacoustic imaging and magnetic resonance) for better control, and they proved that that this system is very efficient in cases of urinary bladder cancer cells in a soft tissue-mimicking model under ex vivo conditions. The development of therapy by immunobots is, therefore, very promising, as many cancer researchers are endeavoring to enhance the accuracy and precision of therapy. The possible effect of MFs on macrophages is demonstrated in the diagram below (Figure 4).

### 3.3. Electroporation Effects on Macrophages

**Figure 5:**
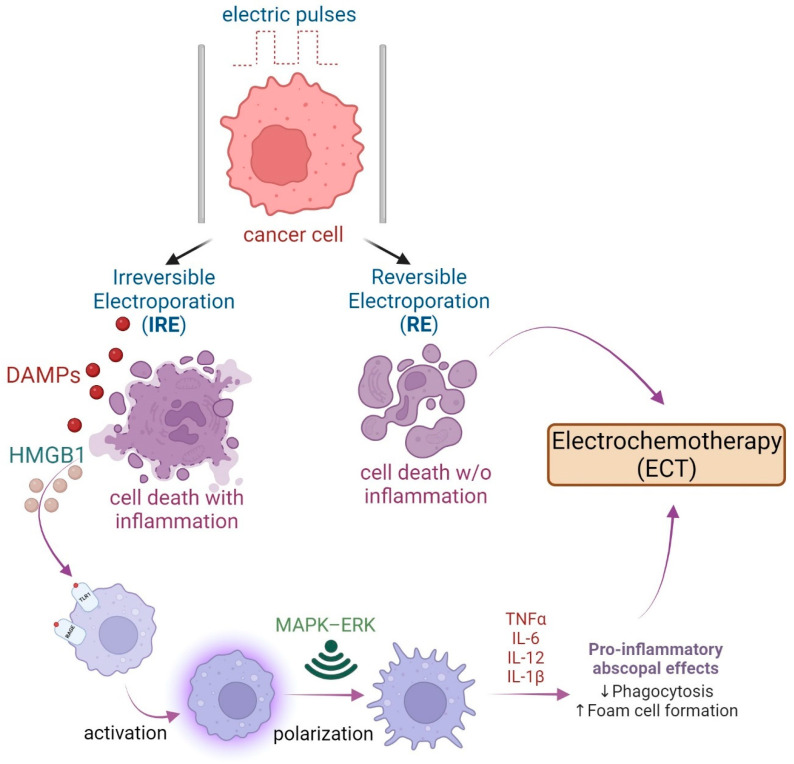
Scheme of the impact of electric pulses on cancer cells, leading to either Irreversible Electroporation (IRE) or Reversible Electroporation (RE). IRE results in cell death accompanied by inflammation, as Damage-Associated Molecular Patterns (DAMPs) and High-Mobility Group Box 1 (HMGB1) proteins trigger the immune response, leading to macrophage activation. This activation proceeds through the MAPK–ERK signaling pathway, resulting in macrophage polarization and a pro-inflammatory state characterized by TNFα, IL-6, IL-12, and IL-1β secretion. These changes can induce abscopal effects, such as reduced phagocytosis and increased foam cell formation. On the other hand, RE results in non-inflammatory cell death and is employed in electrochemotherapy (ECT) to target cancer cells without triggering inflammation.

Although electroporation might not be innovative anymore, its new uses are still gaining interest, especially in the context of tumor fighting and stimulating immune responses [78,79]. For this purpose, electroporation has been developed into a few techniques, including irreversible electroporation (IRE), electrochemotherapy (ECT), and reversible electroporation (EP). One of the advantages of electroporation is that it can be administered in combination with other techniques. For instance, the combination of ECT and IRE with immunotherapy has led to several promising effects [80]. IRE is an ablative technique involving the application of electric pulses (up to 3000 V/cm) to interfere with the tumor cell transmembrane voltage, causing defects that lead to the disruption of cell homeostasis in order to induce cell death [81,82]. Furthermore, it has been noted that IRE exhibits an abscopal (meaning “away from the target”) effect, stimulating the shrinkage of tumors that have not been treated directly [80]. Ablative effects of IRE leave surrounding non-cancerous tissue mostly unaffected, killing tumor cells directly and simultaneously inducing an immune response—immunogenic cell death (ICD) [83]. Although several properties of IRE increase the likelihood of activating the immune system against cancer, the focus of this study is the influence on macrophages. This can be achieved through IRE’s ability to force dying tumor cells to release damage-associated molecular patterns (DAMPs). The increased amount of its main component—high-mobility group box 1 protein (HMGB1)—activates the mitogen-activated protein kinase–p38 (MAPK–p38) pathway by binding to the receptor for advanced glycation end products (RAGE). This results in the reprogramming of tumor-associated macrophages from immune-suppressive (M2) to immune-promoting (M1) phenotypes within the local TME. HMGB1 binding to the RAGE receptor on macrophages further enhances RAGE expression in a positive feedback mechanism and stimulates the autocrine release of HMGB1 in macrophages [83]. Furthermore, HMGB1 promotes changes in the formation of macrophages and enhances adhesive ability and migration, which are helpful with antigen presentation [84]. In addition, M1 macrophage polarization is amplified through the MAPK–extracellular signal-regulated kinase (MAPK–ERK) pathway [83]. Figure 5 shows a schematic representation of the effect of electric pulses on cancer cells and potential macrophage activation.

Studies on the livers of mice suggest that, initially, pro-inflammatory CD11b+ Ly6Chi monocytes are recruited by IRE. Neutrophils also recruited by IRE are believed to promote the conversion of these monocytes to Lys6Clo macrophages. These macrophages have high antigen presentation abilities, leading to increased display of tumor antigens and overcoming resistance to checkpoint inhibitors in pancreatic cancer [85,86]. Phagocytosis by M1 and the resulting antigen presentation are considered to work as an in situ vaccination [87,88]. Furthermore, IRE is suspected to protect vessels, which is also useful with the increased infiltration of M1 macrophages and other immune cells [89]. This could also be responsible for the fact that neutrophils can accumulate deep within the injury zone after IRE [83]. Another technique is reversible electroporation (EP), which is also a local treatment; however, the pores forming in the cell membrane are resealed after several seconds or minutes, and the cells stay intact [90,91,92]. EP can be applied in combination with calcium treatment or chemotherapy as electrochemotherapy (ECT). ECT is commonly used to treat melanoma lesions and esophageal and colorectal cancers [90]. ECT with bleomycin has been shown to stimulate a systemic immune response by enhancing the release of ecto-calreticulin, ATP, and HMGB1 [93]. Research on melanoma-conditioned macrophages showed that treatment with electroporation reduces their viability, in addition to reducing the presence of CD115+ F4/80hi and CD206+ cells (that exhibit M2-like characteristics), in contrast to CD115lo F4/80mid cells (that display M1-like features) [90]. In studies on TAMs in melanoma models, ETC demonstrated the ability to increase their immunogenic capacity. In a biopsy-verified case, it was proven effective for anticancer treatment and showed an abscopal effect against distant tumors [94].

It is worth mentioning that other promising results have been observed in preclinical trials for combinations involving electroporation, highlighting the significance of exploring its potential.

## 4. Combined TAM Therapies

In previous parts of this article, we explained how TAMs interact with anticancer therapies. Nowadays, therapies are being developed that consider those interactions. Mengjun Li et al. [95] listed combined TAM therapy methods, including those combined with chemotherapy (CSF1 blocking mAb  +  bortezomib/melphalan in multiple myeloma, PF-04136309  +  FOLFIRINOX in pancreatic cancer, emactuzumab  +  paclitaxel in advanced/metastatic solid tumors, and R848  +  oxaliplatin in colorectal cancer). In one study of multiple myeloma in mice, the colony-stimulating factor 1 receptor (CSF1R)-blocking mAbs were paired with bortezomib and melphalan. The study suggested the in vivo occurrence of resensitization of the tumor cells to conventional chemotherapy and increased immune response in the tumor environment. Targeting the CSF1/CSF1R axis in combination with chemotherapy decreased tumor burden and increased overall survival of mice [96]. In another non-randomized human trial on a CCR2 inhibitor in combination with FOLFIRINOX, an assessment of safety, tolerability, and dosage took place. The primary agent—PF-04136309—inhibited the CCR2/CCL2 chemokine axis. The trial included 86 patients with pancreatic ductal adenocarcinoma, 39 of whom received the combination treatment. The authors stated that the combination is safe and tolerable [97]. Gomez-Roca C A et al. [98] led a phase I study combining emactuzumab, a monoclonal antibody targeting the CSF-1R, with paclitaxel. However, they did not report any clinically relevant gain from that approach. In 2020, Zhipeng Liu et al. [99] reported the significant promise of utilizing TLR7/8 agonists as a novel boost in chemotherapy for colorectal cancer that is resistant to oxaliplatin. Combinations of TAMs with immunotherapy have also been proposed. CCR2i  +  anti-PD-1 in cutaneous T-cell lymphoma, CCX872  +  anti-PD-1 in glioblastoma, and lefitolimod  +  anti-PD-1 in melanoma are among such examples [95]. Cendrowicz et al. [100] listed other therapies proposed for the targeting of TAMs. A few CSF-1R inhibitors are being investigated. Pexidartinib has already been approved by the FDA for synovial giant cell tumors and has been studied for the treatment of advanced solid tumors. It can also be safely distributed with sirolimus, a combination that presents clinical benefit in 67% of evaluable subjects [101]. One study also treated two patients with gastrointestinal stromal cancer, and meaningful clinical observations were reported, with the therapy well tolerated [102]. ARRY-382 and DCC-3014 have been tested in phase 1 and 2 trials for advanced tumors [100]. Both these agents are potent inhibitors exhibiting structural stability and affinity [103]. ARRY-382 was combined with anti-PD-1 pembrolizumab in a phase 1b/2 study, although limited clinical benefit were observed [104]. Pembrolizumab and chemotherapy were also combined with CXCR4 antagonist BL-9040; phase II trials are ongoing, although this combination therapy has been found to be tolerable and efficient in fighting metastatic pancreatic adenocarcinoma [100]. AMG820 mAb has also been proposed and studied in 1/2 phase monotherapy and in combination with pembrolizumab for advanced solid tumors. This combination showed tolerable toxicity, as well as moderate efficiency [103]. Trabectedin has been approved for the treatment of liposarcoma and leiomyosarcoma, and combination therapies for ovarian cancer are being studied [100]. Zhang et al. [105] made an effort to assess how TAMs may influence the therapeutic effects of ICIs, specifically antiPD-1/PD-L1. It was clear that the role of TAMs in tumor development was significant in both suppressing and boosting growth. Through many signaling pathways, depending largely on tumor type, TAMs up- and downregulate PD-1/PD-L1 expression, which, in turn, takes its toll on the efficacy of PD-1/PD-L1 inhibitors [105]. Tichet et al. [106] claimed that the combination of anti-PD-L1 and PD1-IL2v extended the duration of the response phase in immunotherapy-resistant pancreatic neuroendocrine cancer. In this study of mice PD1-IL2v, an immunocytokine was engineered, combining CD8+ T-cell-targeting PD-1 binding with an IL-2 variant (IL2v) defective in binding to CD25, amplifying regulatory T lymphocytes while directly targeting the tumor. In comparison with sole PD1-IL2v treatment, where 60% of tumors relapsed within 16 weeks, and anti-PD-L1 monotherapy, which presented no significant treatment benefit, the combination therapy resulted in no relapse in 90% of the treated mice in the same observation period. The results present this combinatorial treatment as synergistic. Promoting CD8+ T cells and remodeling immunosuppressive TAMs and vasculature triggers extensive immunological antitumor responses [106].

Another approach proposed by Chryplewicz et al. [107] addresses the poor response of Glioblastoma (GBM) to current systemic therapies. The combination includes imipramine—a tricyclic antidepressant—which reprograms TAMs to immunostimulatory profiles by inhibiting histamine receptor signaling. B20S, a bevacizumab analog, was used as anti-VEGF, as the latter is a clinically approved antibody for the treatment of GBM (mainly for the purpose of reducing brain edema). Anti-VEGF antibodies both remodel vasculature around the tumor and increase the influx of T cells. This combinatorial treatment was reported to have significantly delayed tumor progression and overall survival in the studied mouse model [107]. Many other trials testing the combination of TAM-targeted therapy with immunotherapy have been reported [103]. Another approach to TAM-targeted treatment involves enhancing drug delivery by merging it with artificially engineered nanoparticles. Therapies combining TAMs with nanotechnology are being developed, such as the combination of R848  +  β-cyclodextrin and MiR155  +  LDH in colorectal cancer. In a study on mice, agonist of toll-like receptors (TLRs) TLR7 and TLR8 showed efficient distribution to TAMs when loaded onto β-cyclodextrin nanoparticles (CDNPs). CDNPs richly accumulate tumor tissue—more so than any other examined organ, including the liver, heart, and muscle. Macrophages of the TME were found to shift towards the M1 profile [54]. Moreover, a combination therapy with an immune checkpoint inhibitor—anti-PD-1—was found to be synergistic, resulting in better tumor control and regression. The same approach used in B16.F10 melanoma, otherwise known as anti-PD-1-resistant, showed similar results. This suggests the ability of TAM-associated drugs to potentiate checkpoint inhibitor responses [95]. Nanocarriers with mRNA encoding the M1-like TAM-polarized transcription factor can be used to target mannose receptors of M2 in order to reprogram M2-like TAMs. Layered double hydroxide containing miR155 is another nanoparticle that has the ability to specifically target M2-like TAMs and improves the TME by upregulating TNF-α and IL-12 and co-stimulating CD40, CD80, CD86, and MHC class II [95]. Although stronger therapeutic efficacy is observed when using NPs rather than free drugs, their further application is limited due to low specificity, which causes inefficiency. However, this is an important direction in developing anticancer therapies, multiple forms of nanomedicine (such as Doxil and Abraxane) are already being used in the field [108].

## 5. Summary

Tumor-associated macrophages (TAMs) play a pivotal role in cancer initiation, progression, and metastasis. With growing resistance to conventional cancer therapies, researchers are exploring strategies to modulate TAMs to achieve antitumor effects. Recent studies have shown promise in combining TAM-targeted treatments with chemotherapy, immunotherapy, and nanotechnology. These combinations work synergistically, enhancing drug delivery, improving therapeutic efficacy, and promoting a tumor-suppressive tumor microenvironment (TME).

## Figures and Tables

**Table 1:** List of clinical trials on CAR-Ms in tumors.

ClinicalTrials.gov No.	Title	Target	Drug Agent	Study Type	Result	Study Start and Finish
NCT06224738	Human HER2-targeted Macrophages Therapy for HER2-positive Advanced Gastric Cancer With Peritoneal	HER2	human HER2 CAR-M	Early phase 1	No results	March 2024–March 2026
NCT04660929	CAR-macrophages for the Treatment of HER2 Overexpressing Solid Tumors	HER2	CT-0508	Phase 1	No results	February 2021–February 2024
NCT03608618	Intraperitoneal MCY-M11 (Mesothelin-targeting CAR) for Treatment of Advanced Ovarian Cancer and Peritoneal Mesothelioma	Mesothelin	Intraperitoneal MCY-M11 and cyclophosphamide	Phase 1	No results	August 2018–August 2021
NCT05007379	Cohort Study to Determine the Antitumor Activity of New CAR-macrophages in Breast Cancer Patients’ Derived Organoids (CARMA)	HER2	HER2 CAR-M	Observational	No results	September 2021–September 2023

**Table 2:** Abilities, advantages, and problems associated with CAR-M therapy.

CAR-M Abilities	CAR-M Advantages	CAR-M Therapy Problems	Refs
Phagocytosis of selective antigen-expressing tumor cells	Low GvHD risk	Limited efficacy in CAR transduction	[41,56,57,64]
Migration and infiltration into the immunosuppressive tumor environment	Resistance to the effects of immunosuppressive cytokines	Requires differentiation into the M1 antitumor phenotype	
Induction of phenotypic shifts in M2 macrophages to the M1 type	Important source of matrix metalloproteinase (MMP), which degrades almost the entire ECM (extracellular matrix)	Could lead to a cytokine storm, CRS, and off-target toxicity	
Activation of T cells	Ability to overcome physical barriers within the tumor microenvironment	Potential challenges in achieving adequate CAR-M cell numbers for effective therapy	
Presentation of tumor antigens to Th1 cells	Enhanced antigen presentation due to increased expression of costimulatory molecules	High cost and complexity of manufacturing CAR-M cells	
Recruitment of leukocytes	Potential for combination with other therapies, such as checkpoint inhibitors, for synergistic effects	Limited in vivo persistence of CAR-M cells after infusion	
Induction of adaptive immune responses and production of inflammatory factors	Longevity and persistence in the tumor microenvironment, leading to sustained antitumor activity	Risk of unintended effects on non-tumor cells, potentially leading to autoimmunity or other adverse events	

## Data Availability

Not applicable.
